# The structure of a potassium-selective ion channel reveals a hydrophobic gate regulating ion permeation

**DOI:** 10.1107/S2052252520008271

**Published:** 2020-07-25

**Authors:** Patricia S. Langan, Venu Gopal Vandavasi, Wojciech Kopec, Brendan Sullivan, Pavel V. Afonne, Kevin L. Weiss, Bert L. de Groot, Leighton Coates

**Affiliations:** a Neutron Scattering Division, Oak Ridge National Laboratory, 1 Bethel Valley Road, Oak Ridge, TN 37831, USA; b Lentigen Technologies, 910 Clopper Road, Gaithersburg, MD 20878, USA; c Department of Chemistry, Princeton University, Princeton, NJ 08540, USA; d Biomolecular Dynamics Group, Max Planck Institute for Biophysical Chemistry, 37077 Göttingen, Germany; e Molecular Biophysics and Integrated Bioimaging Division, Lawrence Berkeley National Laboratory, Berkeley, CA 94720, USA

**Keywords:** potassium ion channels, gating, X-ray crystallography, conformational transitions

## Abstract

Protein dynamics are essential to function. One example of this is the various gating mechanisms within ion channels, which are transmembrane proteins that act as gateways into the cell. Typical ion channels switch between an open and closed state via a conformational transition which is often triggered by an external stimulus, such as ligand binding or pH and voltage differences. The atomic resolution structure of a potassium-selective ion channel named NaK2K has allowed us to observe that a hydro­phobic residue at the bottom of the selectivity filter, Phe92, appears in dual conformations. One of the two conformations of Phe92 restricts the diameter of the exit pore around the selectivity filter, limiting ion flow through the channel, while the other conformation of Phe92 provides a larger-diameter exit pore from the selectivity filter. Thus, it can be concluded that Phe92 acts as a hydro­phobic gate, regulating the flow of ions through the selectivity filter.

## Abstract 2

The atomic resolution structure of a potassium-selective ion channel reveals a hydrophobic gate that regulates ion permeation.

## 1. Introduction   

Ion channels are a physiologically essential class of proteins that are ubiquitously expressed in organisms ranging from bacteria to human beings (Roux, 2017 ▸). They reside within cellular membranes and the lipid bilayers that segment cellular compartments, where they provide a barrier to the passage of components, which is vital for an array of physiological processes. Potassium ion channels are essential elements in cellular electrical excitability (Hille, 2001 ▸), while also maintaining a resting potential in non-excitable cells (Miller, 2001 ▸). Inside the cell, the concentration of potassium ions is >100 m*M*, while on the outside of the cell, the potassium ion concentration is usually <5 m*M* (Zhou *et al.*, 2001 ▸).

The structural determination of several potassium ion channels has increased our understanding of potassium ion permeation, which underlies electrical signaling in cells (Doyle *et al.*, 1998 ▸; Jiang *et al.*, 2002*a* ▸; Alam & Jiang, 2009*a* ▸). The signature sequence in potassium ion selectivity is the TVGYG sequence which forms the selectivity filter of the K^+^ ion channel (Doyle *et al.*, 1998 ▸; Heginbotham *et al.*, 1992 ▸). The selectivity filter displays a high selectivity for potassium ions over sodium ions and is formed from four contiguous and chemically equivalent binding sites (Doyle *et al.*, 1998 ▸).

The protein’s three-dimensional structure orients around 20 carbonyl oxygen atoms into the ion-channel pore, each with a partial negative charge. This would make the selectivity filter an unstable structure was it not for the presence of positive potassium ions within the selectivity filter. The first structure of a potassium ion channel was solved from the bacteria *Streptomyces lividans* and was named KcsA. In the KcsA potassium ion channel, the oxygen to potassium ion coordination distances range from 2.70 to 3.08 Å with a mean value of 2.85 Å (Zhou *et al.*, 2001 ▸), while a network of hydrogen bonds involving the amide nitro­gen atoms of the amino acid residues that make up the selectivity filter stabilize the structure of the ion channel.

The TVGDG selectivity filter sequence of the NaK channel from *Bacillius cerus* differs from the conserved TVGYG selectivity filter sequence found in potassium ion channels by a single amino acid substitution of Tyr to Asp. This helps to alter the conformation of the selectivity filter, forming a non-selective specificity filter which allows both potassium and sodium ions to be permeated through the ion channel (Sauer *et al.*, 2013 ▸). The NaK channel shares a high amino acid sequence homology and a similar structure (Shi *et al.*, 2006 ▸) with the bacterial KcsA potassium ion channel (Doyle *et al.*, 1998 ▸; Zhou *et al.*, 2001 ▸) from *S. lividans*, but its selectivity filter adopts a different conformation (Shi *et al.*, 2006 ▸). Both NaK and KcsA are tetrameric ion channels composed of four identical polypeptide chains which form a symmetrical structure. At their center is a pore lined with the conserved residues which form the selectivity filter. Crystallization of the full-length amino acid sequence of NaK indicated that the first 19 amino acids at the N-terminal end of the protein form an interfacial helix, often called the M0 helix, which is parallel to the cellular membrane. The four M0 helices in the tetrameric NaK structure encircle the inner helix bundle, which has been proposed to affect the opening and closing of the ion-channel pore and lock the structure into a closed form (Shi *et al.*, 2006 ▸). Truncating the construct to remove the N-terminal M0 helix places the NaK channel in an ‘open’ form as the inner helices twist around a flexible glycine residue (Gly87) placing NaK in an open form. Gly87 is known to act as a gating hinge (Alam & Jiang, 2009*a* ▸; Jiang *et al.*, 2002*b* ▸) in other tetrameric ion channels such as MthK, a calcium-gated potassium ion channel (Jiang *et al.*, 2002*a* ▸) from *Methano­bacterium thermoautotrophicum*.

The deletion of the M0 helix also reduces the volume of the unit cell, changes the space group and significantly increases the diffraction resolution of data which can be collected on the NaK crystals from 2.40 to 1.80 Å (Alam & Jiang, 2009*a* ▸; Shi *et al.*, 2006 ▸). This deletion also produces higher ion-permeation rates as measured using the time-dependent accumulation of ^86^Rb inside liposomes (Shi *et al.*, 2006 ▸). After an incubation time of 10 min, ^86^Rb ion flux is four times higher using truncated NaK compared with using the full-length NaK protein (Shi *et al.*, 2006 ▸). Later crystallographic studies (Alam & Jiang, 2009*a* ▸) on the truncated form of NaK indicated that Phe92 might be a possible constriction point in this open form of the ion channel. This was confirmed by the Phe92Ala mutant showing an increase in ion-conduction rates for ^86^Rb flux into liposomes by a factor of 12 after an incubation time of 10 min (Alam & Jiang, 2009*a* ▸), showing that Phe92 plays a vital role in the regulation of ion permeation through the selectivity filter while it is in the open form.

### 1.1. The NaK2K potassium-selective mutant   

**Figure 1:**
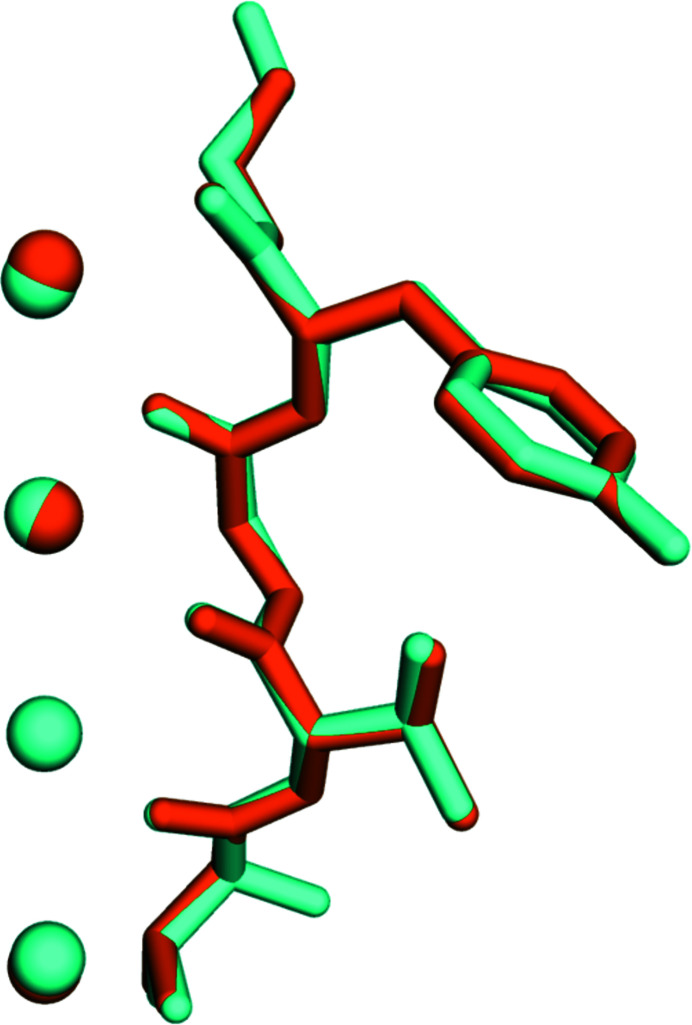
The KcsA amino acid residues that form the selectivity filter, Thr75 to Gly79, and four potassium ions are shown in cyan, while residues Thr63 to Gly67, which form the NaK2K selectivity filter, and four potassium ions are shown in orange. The main-chain RMSD for these residues is only 0.12 Å, indicating that the selectivity filters are identical.

The introduction of Asp66Tyr and Asn68Asp mutations in the NaK amino acid sequence causes main-chain conformational changes of the GYG residues in the selectivity filter (Sauer *et al.*, 2011 ▸). This double NaK mutant named NaK2K (Derebe *et al.*, 2011 ▸) possesses the conserved amino sequence (TVGYG) found in all potassium ion channels. The structure on the selectivity filter in NaK2K is almost identical (a root-mean-square deviation, RMSD, for main-chain atoms of 0.12 Å) to that found in the K^+^ ion channel KcsA (Sauer *et al.*, 2013 ▸) and is highly selective for K^+^ ions (Fig. 1 ▸). This value can be compared with an RMSD value of 1.60 Å between the selectivity filters of NaK and KcsA. Besides these two point mutations, the NaK2K construct we used also has the first 19 amino acids at the N-terminal end of the protein which forms the M0 or interfacial helix removed placing it into the open conformation (Alam & Jiang, 2009*a* ▸).

### 1.2. Permeation of potassium ions   

Crystal structures of the potassium channel from the soil bacteria *S. lividans* (KscA) clearly show the four discrete K^+^ binding sites (Doyle *et al.*, 1998 ▸; Zhou *et al.*, 2001 ▸) within the selectivity filter. However, it was not possible to determine how many of these sites are occupied with ions at any given time as the observed electron density at each binding site is an average value based on all possible states present within the crystal. Anomalous scattering data has been collected from KscA in which K^+^ ions were replaced with thallium ions (Zhou & MacKinnon, 2003 ▸) (Tl^+^) to indirectly determine the total number of K^+^ ions in the filter. The average occupancy at each site within the selectivity filter was 0.63 which inferred an occupancy of 0.53 for K^+^ ions and therefore the total number of potassium ions in the selectivity filter was 2. This formed the basis of the widely accepted co-translocation mechanism. Later on, this data was re-examined using both molecular dynamics (MD) and protein crystallography to reveal that all four binding sites in the selectivity filter are fully occupied with potassium (Köpfer *et al.*, 2014 ▸).

Recently, with the construction of dedicated long-wavelength X-ray beamlines such as I23 at Diamond Light Source (Wagner *et al.*, 2016 ▸), it finally became possible to collect X-ray data at wavelengths corresponding to the anomalous scattering edge of potassium. In an earlier study, we collected anomalous scattering data from NaK2K at the potassium adsorption edge which also showed that the selectivity filter is fully occupied with four potassium ions (Langan *et al.*, 2018 ▸). This result is in agreement with several recent studies which re-examined crystallographic data (Köpfer *et al.*, 2014 ▸), MD simulations of ion flow through the selectivity filter (Kopec *et al.*, 2018 ▸) and, most recently, hydrogen/deuterium exchange rates as monitored by NMR (Öster *et al.*, 2019 ▸). These simulations indicate that the strong electrostatic interactions between tightly packed potassium ions within the selectivity filter play a key role in generating the high rates of potassium ion conductance. Because of the longer X-ray wavelengths (3.35 Å) that were used to collect the anomalous data, the resolution of this structure was limited to ∼2.26 Å. Using much shorter wavelength X-rays (0.97 Å), we were able to collect a dataset to atomic resolution (1.20 Å) for NaK2K, enabling us to reveal in unprecedented detail the protein structure of NaK2K.

## 2. Results and discussion   

To date, few membrane protein structures have been determined at atomic resolution (1.20 Å) or better (Kosinska Eriksson *et al.*, 2013 ▸), during refinement discrete features of the structure of NaK2K were elucidated that were not observed in the original 1.55 Å resolution structure (Sauer *et al.*, 2011 ▸). The atomic resolution dataset enabled us to conduct anisotropic *B*-factor refinement on all atoms within the protein structure and to add hydrogen atoms onto the protein structure during refinement. Riding hydrogen atoms were added to the model to better account for the electron density and to limit clashes between amino acid sidechains.

This produced an extremely accurate and well refined structure with an *R*_factor_ and *R*_free_ of 13.26 and 14.77%, respectively, compared with the higher *R*_factor_ and *R*_free_ values of 20.00 and 21.50% reported for the original structure. Our structure possesses excellent stereochemistry with a molprobity (Chen *et al.*, 2010 ▸) clash score of 2, with no sidechain or Ramachandran outliers. This compares favorably with the original structure which has a molprobity clash score of 8, with 0.5 and 1.2% of the residues listed as Ramachandran and/or sidechain outliers, respectively.

The overall protein structure of NaK2K remains similar to the previously reported structure (a RMSD of 0.28 Å for all C_α_ atoms). However, the increased resolution of our structure enabled us to visualize several unseen alternative conformations, such as Phe92, which has an important role in regulating the flow of ions through the selectivity filter. During refinement, we carefully analyzed the hydrated potassium ions that were modeled into the original structure (Sauer *et al.*, 2011 ▸). These atoms are modeled just above the selectivity filter close to Tyr66, and electron density is also present in our structure at this position. However, when we inspected the anomalous difference map from our earlier work on NaK2K (Langan *et al.*, 2018 ▸), we saw no anomalous difference peak at this position, indicating that the atom at this position is probably not a potassium ion as modeled in the original structure. The identity of the atom(s) at this position is unknown and speculative at best. Therefore, we left them unmodelled in our structure. We also observed some partial electron density for two partially occupied and highly disordered *n*-Decyl-β-d-Malto­pyran­oside (DM) molecules which could not be satisfactorily modeled into the structure.

### 2.1. Dual conformations of Phe92 suggest a role in ion-channel gating   

**Figure 2:**
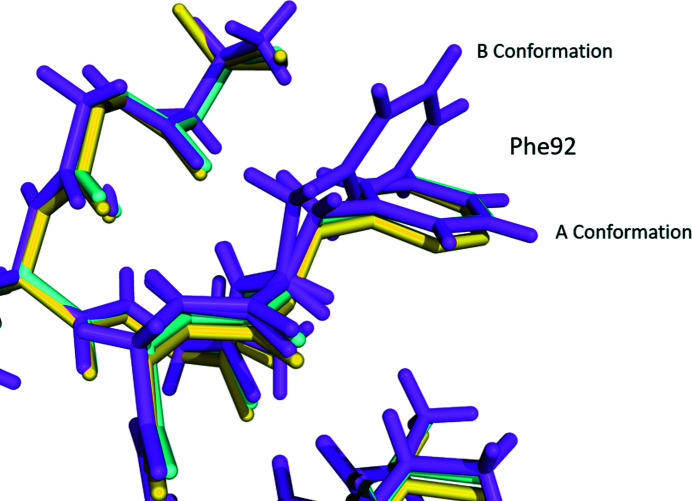
An overlay of the various conformations of Phe92 seen in the structure of the open form of NaK, colored yellow (PDB code 3e8h; Alam & Jiang, 2009*b* ▸); the open form of NaK2K, colored cyan (PDB code 3ouf; Derebe *et al.*, 2011 ▸); and our atomic resolution structure of the open form of NaK2K, colored magenta (PDB code 6ufe).

Within the asymmetric unit of the crystal are two identical polypeptide chains (A and B). When the fourfold symmetry of the *I*4 space group is applied, three additional symmetry-related molecules are generated to form an intact potassium ion channel from each of the two polypeptide chains in the asymmetric unit. In the A polypeptide chain, at the bottom of the selectivity filter on the intracellular side Phe92 is observed in a dual conformation, with the A conformation having a refined occupancy of 68% and the B conformation having a refined occupancy of 32%. In the B polypeptide chain within the unit cell, Phe92 is again in dual conformation, with a refined occupancy of 69% for the A conformation and 31% for the B conformation. Phe92 in previous NaK2K (Sauer *et al.*, 2011 ▸) and NaK structures (Alam & Jiang, 2009*a* ▸) was modeled in a single conformation that corresponds to the A conformation seen in our structure (Fig. 2 ▸).

To confirm the presence of both conformers of Phe92, we used polder OMIT maps (Liebschner *et al.*, 2017 ▸), which are useful for displaying weak density associated with partially occupied or highly mobile atomic moieties, such as ligands, loops or alternative sidechain conformations. Since it has been made available in *Phenix* software (Adams *et al.*, 2010 ▸), this method has been used in 117 documented cases, proving its utility. Polder OMIT maps differ from conventional OMIT maps (Bhat & Cohen, 1984 ▸) in that the electron density associated with the bulk solvent is excluded from the omitted region. A complete description of how polder OMIT maps have been implemented in *Phenix* is given in the work of Liebschner *et al.* (2017 ▸).

**Figure 3:**
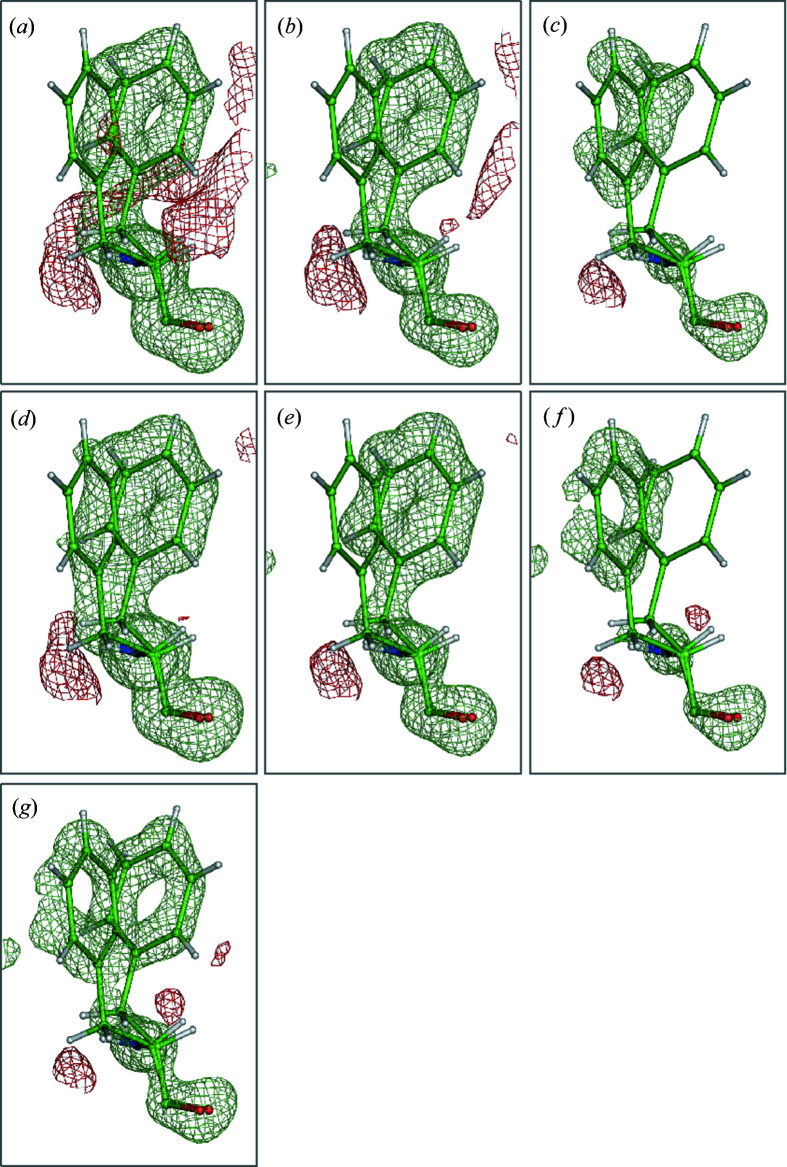
*m* *F*_o_ − *D* *F*_c_ OMIT density maps of Phe92 (chain A) from the structure of a potassium ion channel at atomic resolution contoured at a level of 2.6σ. Panels (*a*), (*b*) and (*c*) show conventional OMIT maps, while panels (*d*), (*e*) and (*f*) show polder OMIT maps. In panels (*a*) and (*d*), both conformations of Phe92 have been omitted; in panels (*b*) and (*e*), the A conformation of Phe92 has been omitted; and in panels (*c*) and (*f*), the B conformation of Phe92 has been omitted. In panel (*g*), a composite polder OMIT map is shown.

Here we describe a new procedure to calculate a polder OMIT map for cases when the atoms in question belong to different conformations. The procedure begins with analyzing atoms to OMIT and then grouping them by alternative conformations. Then each group of atoms is omitted, one at a time, and the conventional *m* *F*_o_ − *D* *F*_c_ polder map is calculated and stored. Once all the maps corresponding to all the atom groups are calculated, they are scaled together locally in the OMIT region. The final polder composite OMIT map (Fig. 3 ▸) is assembled by assigning the maximum contribution to the given grid node in the composite map calculated from corresponding grid nodes from all the individual maps. A map calculated in this way equalizes the signal that arises from each conformation while not biasing the whole map by the bulk-solvent contribution. We found that this procedure was highly beneficial when looking at amino acid sidechains in multiple conformations that overlap with each other and are not equally occupied. This is probably because of two reasons. Firstly, one of the conformations was substantially more populated than the other (occupancy = 0.68 versus 0.32), and secondly, both conformations are located sufficiently close to each other so the density from the more-occupied conformation would dominate and obscure the conformation that is less occupied.

We utilized the procedure detailed above to generate a composite polder OMIT map [Fig. 3 ▸(*g*)] to represent the polder OMIT density for each conformation of Phe92 within a single composite polder OMIT map. This procedure generated a more convincing OMIT map (compared with ordinary OMIT or polder maps), confirming the presence of both conformations of Phe92, free from atomic overlaps and bulk-solvent effects [Fig. 3 ▸(*g*)].

**Figure 4:**
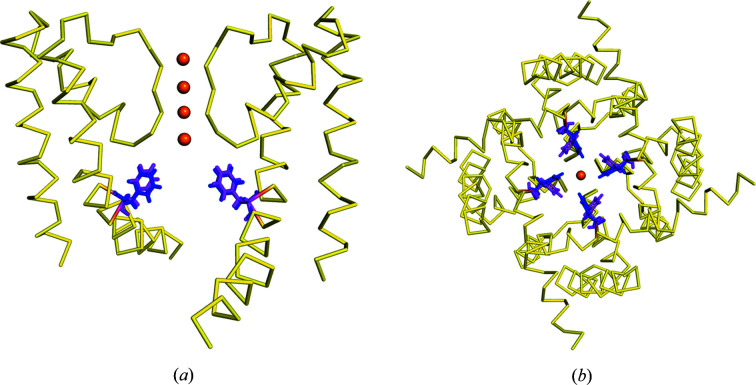
The structure of the NaK2K ion channel is shown as ribbons with the residue Phe92 shown as sticks. The A conformation of Phe92 is shown in magenta while the B conformation of Phe92 is shown in blue, with potassium ions shown as orange spheres. (*a*) Phe92 is located at the bottom of the selectivity filter, for clarity only two of the four subunits that form a complete ion channel are shown. (*b*) A bottom-up view of a complete ion channel, again conformation A of Phe92 is shown in magenta while the B conformation of Phe92 is shown in blue.

Phe92 acts as a mobile restriction point at the bottom of the ion channel that limits ion flow through the channel and plays an important part in the regulation of ion conduction (Fig. 4 ▸).

**Figure 5:**
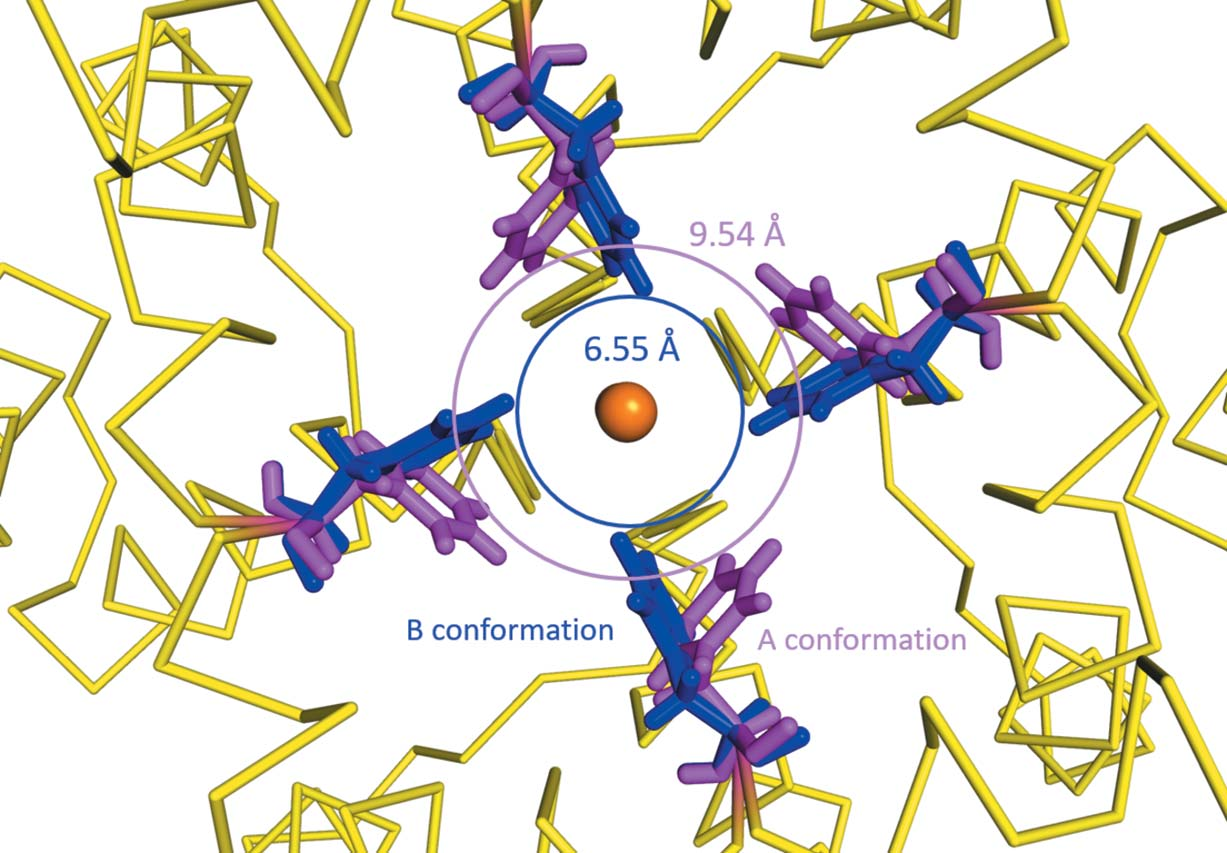
The A and B conformations of Phe92 observed in our structure create pores with different diameters at the exit of the selectivity filter. The A conformation of Phe92 shown in magenta creates a pore with a diameter of 9.54 Å, which we refer to as the enlarged pore. The B conformation of Phe92 shown in blue creates a pore with a diameter of 6.55 Å, which we refer to as the restricted pore. We are defining the pore diameter as the shortest distance between atoms on opposing Phe92 residues in the ion channel based upon our crystal structure.

As previously mentioned, the mutation of Phe92 to Ala significantly increases ionic flux through the channel (Sauer *et al.*, 2011 ▸). Experiments using a Phe92Ala mutant of NaK increased the influx of ^86^Rb into KCl-loaded liposomes by a factor of 12 after an incubation time of 10 min (Alam & Jiang, 2009*a* ▸). The dual conformations of Phe92 seen in our structure offer a structural explanation for this biochemical observation (Alam & Jiang, 2009*a* ▸). In other cation channels, the residue equivalent to Phe92 is often replaced with a smaller amino acid sidechain (Alam & Jiang, 2009*a* ▸), such as alanine in MthK (Jiang *et al.*, 2002*a* ▸), which then forms the narrowest part of this ion channel. The distance between the CE atoms of both conformations of Phe92 in our structure is 2.97 Å, with the B conformation restricting the size of the pore at the exit of the selectivity filter to 6.55 Å compared with a pore diameter of 9.54 Å for the more-occupied A conformation (Fig. 5 ▸). The A conformation (68% occupancy) corresponds to an enlarged pore conformation of the ion channel in its open form while the B conformation (32% occupancy) corresponds to a restricted form of the open form of the ion channel. We define the pore diameter as the shortest distance between atoms on opposing Phe92 residues in the ion channel based upon our crystal structure.

Thus, the dual conformations of Phe92 observed in our structure indicate that in the open form of NaK2K, which is caused by the deletion of the interfacial helix, the two conformations of Phe92 co-occur; one is a restricted pore form which limits ion conductance while the other is an enlarged pore form which is more conducive to ion permeation. In the open-form structure (Jiang *et al.*, 2002*b* ▸) of NaK, the pore diameter defined by Phe92 and measured from the crystal structure (Protein Data Bank, PDB code 3e86; Alam & Jiang, 2009*a* ▸) is 9.25 Å with hydrogen atoms added to the model, a value that is very similar to the 9.54 Å pore diameter observed in the enlarged pore A conformation of Phe92 seen in our structure.

**Figure 6:**
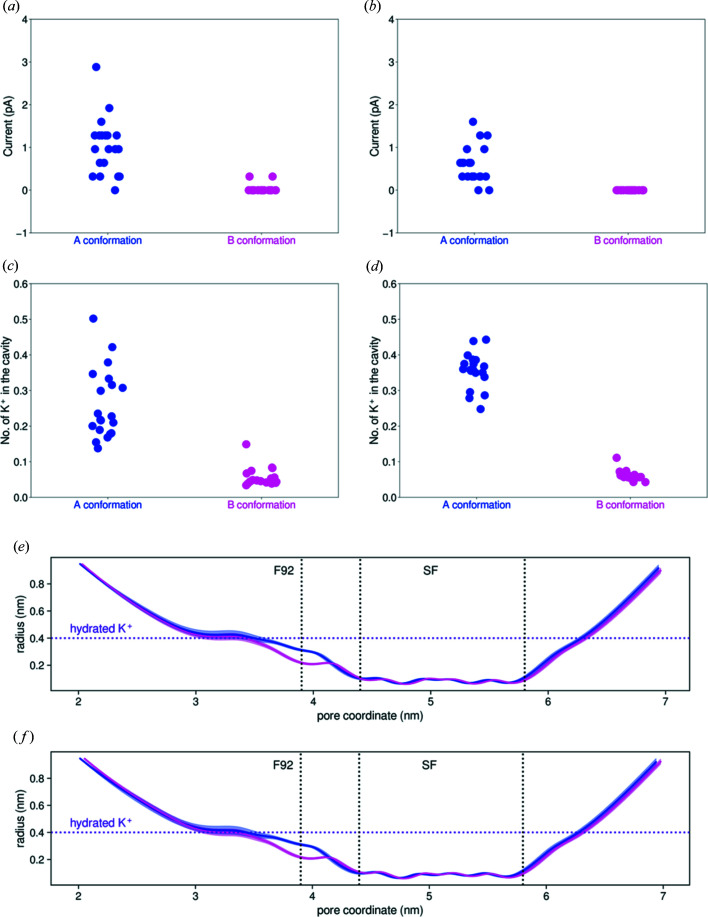
MD simulations of A (blue) and B (magenta) conformations of NaK2K. (*a*), (*b*) Ionic currents through NaK2K at the membrane voltages of 300 (*a*) and −300 mV (*b*). Each dot represents an independent 500 ns long MD simulation. (*c*), (*d*) An average number of potassium ions found in the cavity (ions within 0.6 nm of the center of mass of Phe92 were considered), at 300 and −300 mV, respectively. (*e*), (*f*) Pore radius profiles for simulations at 300 and −300 mV, respectively. The horizontal purple line marks the typical radius of a fully hydrated potassium ion. The average position of the Phe92 aromatic ring is also indicated, as well as the selectivity filter. The error bars indicate 95% confidence intervals.

To directly probe the impact of the Phe92 conformation on the ion flux through NaK2K, we performed MD simulations under applied voltage, using both the A and B conformations (see Materials and methods[Sec sec4]). Such an approach provides a clear view into the details of the ion-permeation process on the atomistic scale and has been recently used to elucidate the mechanisms of ion permeation (Köpfer *et al.*, 2014 ▸), selectivity (Kopec *et al.*, 2018 ▸) and gating (Kopec *et al.*, 2019 ▸) in potassium channels. At both positive and negative voltages, we observe ionic currents in each simulation, arising from ions permeating the channel, when NaK2K assumes the A conformation [Figs. 6 ▸(*a*) and 6 ▸(*b*]. In contrast, and in full agreement with predictions made based on the crystal structure, the B conformation is not compatible with ion permeation in both directions. To obtain further insight into why this is the case, we calculated the average number of potassium ions in simulations in a cavity region [Figs. 6 ▸(*c*) and 6 ▸(*d*]. While potassium ions are able to access the cavity in the A conformation of NaK2K and then continue to the selectivity filter, the smaller cavity formed in the B conformation restricts the access of potassium ions and consequently they are very rarely found in the cavity (the average occupancy of this region is below 0.1). The narrowing of the cavity in the B conformation, observed in the crystal structure, is seen as well in MD simulations [Figs. 6 ▸(*e*) and 6 ▸(*f*].

### 2.2. Potassium ion occupancy within the selectivity filter   

Within the selectivity filter, there are four discrete binding sites; we used our atomic resolution structure to further verify and examine the potassium ion occupancy at each of the four binding sites. The results of the occupancy refinements conducted in *Phenix* are given in Table 1 ▸.

As can be seen from Table 1 ▸, the mean potassium occupancy value for chains A and B is 0.26. These data are in agreement with recent studies that re-examined existing X-ray data and used MD and NMR to investigate potassium ion permeation and the occupancy of potassium ions in the selectivity filter (Köpfer *et al.*, 2014 ▸; Kopec *et al.*, 2018 ▸; Öster *et al.*, 2019 ▸). All of these techniques concluded that each of the four binding sites within the selectivity filter of NaK2K were fully occupied with potassium ions and that ion permeation occurs via direct coulomb knock on.

## 3. Conclusions   

Using our NaK2K atomic resolution dataset, we have been able to gain new insights into the role that Phe92 plays in regulating ion flow by being able to move between two conformations. The enlarged pore form enables potassium ions to exit the selectivity filter into the cell and has a diameter that closely matches that seen in an open conformation of NaK, while the restricted pore form probably limits ion flow through the selectivity filter into the cell. This explains previous biochemical results which showed an increase in ion-conduction rates of a factor of 12 for ^86^Rb flux into liposomes after an incubation time of 10 min for the Phe92Ala mutant. The transition between the two conformations of Phe92 could be driven by the presence or absence of a hydrated potassium ion at the exit of the selectivity filter. This would explain the role of Phe92 in regulating the permeation of potassium ions through the selectivity filter.

Interestingly, sidechain rearrangement of Phe103 in KcsA, which is also located at the bottom of the selectivity filter, has been proposed to mechanically couple activation and inactivation in KcsA and other potassium ion channels (Pan *et al.*, 2011 ▸; Zhou & McCammon, 2010 ▸). While the restricted form closes the bottom of the selectivity filter restricting ion flow into the cell, large aromatic residues in other potassium ion channel structures such as Phe146 in KirBac1.1 (Kuo *et al.*, 2003 ▸) and Tyr132 in KirBac3.1 (Kuo *et al.*, 2005 ▸) have also been proposed to act as gates. While Phe203 and Tyr215 play similar roles in MlotiK1, substituting these residues for Ala also leads to significant increases in the rate of ion uptake (Clayton *et al.*, 2008 ▸), as has been seen for NaK (Shi *et al.*, 2006 ▸; Alam & Jiang, 2009*a* ▸). Thus, we conclude that the hydro­phobic residue Phe92 in NaK/NaK2K acts as a hydro­phobic gate that regulates ion permeation across the cellular membrane.

## 4. Materials and methods   

### 4.1. Protein purification   

A plasmid containing the NaK mutant NaK2K from *Bacillus cereus* m1550 in the pD441 vector was purchased from ATUM and transformed into *Escherichia coli* BL21 competent cells. This construct did not contain the first 19 amino acid residues which form the M0 helix but contained the Asp66Tyr and Asn68Asp mutations. Cultures were inoculated by scraping colonies from transformation plates into LB media, grown at 37°C, and induced at A600 0.6 with 0.4 m*M* IPTG for 18 h at 25°C. Cells were then pelleted by centrifugation. For every gram of cells (wet weight), 5 ml of lysis buffer (50 m*M* Tris pH 7.8, 100 m*M* KCl), SIGMAFAST protease inhibitor tablets (Millipore Sigma) and 1 mg ml^−1^ of lysozyme were added. Cells were resuspended by slow stirring at room temperature for 30 min and were then further lysed by sonication. Cell debris was removed by centrifugation at 10 000*g* and NaK2K was solubilized by incubation of the supernatant at room temperature for 2 h with 40 m*M* Sol-grade DM. Additional debris was removed from the lysate by centrifugation at 21 000*g* for 30 min. The protein was then purified on a TALON metal affinity resin using buffers containing 4 m*M* DM. Fractions containing NaK2K were pooled, and the 6XHis tag was removed by adding 1 unit of thrombin per 1 mg of NaK2K and incubating at room temperature for 16 h. NaK2K was then concentrated using a 30 kDa molecular weight cut-off (MWCO) concentrator and further purified on a Superdex 200 increase 10/300 GL column using 20 m*M* Tris–HCl pH 7.8, 100 m*M* KCl and 4 m*M* Anagrade DM.

### 4.2. Protein crystallization   

A solution of purified NaK2K protein solution was concentrated to 14 mg ml^−1^ using a 50 kDa MWCO concentrator. The crystals used for data collection were grown in sitting drops prepared by mixing equal volumes of protein solution in a buffer (20 m*M* Tris–HCl pH 7.8, 200 m*M* KCl and 4 m*M* Anagrade DM) with well solution (72.5% MPD, 100 m*M* KCl and 200 m*M* MES pH 6). The crystal was flash frozen in liquid nitro­gen with no further cryoprotectant being added.

### 4.3. Atomic resolution 100 K X-ray data-collection processing and refinement   

X-ray diffraction data were collected at 100 K over a range of 120° (0.5° steps) using an ADSC Quantum 315r detector at the Advanced Photon Source (APS) on the ID19 beamline SBC-CAT to 1.20 Å resolution. A high-resolution pass was collected first with a detector distance of 150 mm, this was followed by the collection of a low-resolution pass with a detector distance of 300 mm over the same φ range. The crystal used for data collection was flash frozen in liquid nitro­gen with no further cryoprotectant being added. All data were processed and reduced using *XDS* (Kabsch, 2010 ▸) and *XSCALE*, while model building and refinement were conducting using *Coot* (Emsley & Cowtan, 2004 ▸) and *phenix.refine* from the *Phenix* suite (Adams *et al.*, 2010 ▸). The program *Xtriage* from the *Phenix* suite was used to check for signs of twinning, with no twinning being found. The data and refinement statistics are shown in Table 2 ▸.

### 4.4. MD simulations   

The new crystal structures of NaK2K reported in this work were protonated according to the standard protonation states at pH 7, inserted into the POPC membrane (145 lipids), and surrounded by water molecules (∼11 000) and ions (174 K^+^ and 166 Cl^−^), resulting in a salt concentration of ∼1*M*. Crystallographically resolved water molecules and ions were retained. The protein termini were capped with ACE and NME groups. These modeling steps were carried out using the *CHARMM-GUI* webserver (Jo *et al.*, 2008 ▸, 2009 ▸; Lee *et al.*, 2016 ▸; Wu *et al.*, 2014 ▸). Consequently, the systems were equilibrated using scripts provided by *CHARMM-GUI* with *GROMACS* 2019 software (Abraham *et al.*, 2015 ▸; Bjelkmar *et al.*, 2010 ▸; Hess *et al.*, 2008 ▸; Pronk *et al.*, 2013 ▸; Van Der Spoel *et al.*, 2005 ▸). After these initial equilibrations, the systems were converted into the format compatible with the *CHARMM*36m force field (Huang *et al.*, 2017 ▸; Klauda *et al.*, 2010 ▸; MacKerell *et al.*, 1998 ▸) (downloaded from http://mackerell.umaryland.edu/index.shtml) and further equilibrated using position restraints on heavy atoms of Phe92 (with a force constant of 1000 kJ mol^−1^ nm^−2^) for 100 ns, using the *CHARMM*36m force field, the *CHARMM* TIP3P water model and default *CHARMM* ion parameters (Beglov & Roux, 1994 ▸). All bonds containing hydrogen atoms were constrained using a linear constraint solver for molecular simulations (*LINCS*) (Hess *et al.*, 1997 ▸), allowing for a integration step of 2 fs. Van der Waals interactions were force-switched off from 0.8 to 1.2 nm. The PME was used with the 1.2 nm real-space cut-off. The Nosé–Hoover thermostat (Nosé, 1984 ▸; Hoover, 1985 ▸) and Parrinello–Rahman barostat (Parrinello & Rahman, 1981 ▸) were used to keep the simulated systems at 310 K and 1 bar, respectively. After the equilibration, each system was simulated by an external electric field applied along the *z* axis to generate a membrane voltage of ∼300 mV or −300 mV to study ion permeation. In total, four systems were simulated: NaK2K with Phe92 restrained in two conformations (A and B), each at two voltages (300 mV and −300 mV). For each system, 20 individual simulations (500 ns each) were performed, resulting in a total simulation time of 40 µs. We report individual ion permeation events, obtained using a custom *Fortran* code available in our recent publication (Kopec *et al.*, 2019 ▸), the number of ions in the cavity, obtained using the gmx select command of *GROMACS*, and the channel radius profile, obtained using *HOLE* software (Smart *et al.*, 1996 ▸).

## Supplementary Material

PDB reference: Open form of NaK2K, 6ufe

## Figures and Tables

**Table 1:** Refined occupancy values for potassium ions within the selectivity filter Sites A_1_ to A_4_ occur within the A chain while sites B_1_ to B_4_ occur within the B chain. Each potassium ion within the selectivity filter is shared between four unit cells and thus has a maximum occupancy value of 0.25. Occupancy values above 0.25 arise because of the correlation between the atomic displacement parameter and occupancy.

Binding site	Refined occupancy
A_1_	0.24
A_2_	0.25
A_3_	0.25
A_4_	0.28
B_1_	0.25
B_2_	0.22
B_3_	0.28
B_4_	0.29

**Table 2:** Data and refinement statistics for our atomic resolution X-ray structure of the open form of NaK2K The highest-resolution shell is shown in parentheses.

PDB accession code	6ufe
Unit-cell parameters (*a*, *b*, *c*, α, β, γ) (Å, °)	67.86, 67.86, 89.62, α = β = γ = 90
Wavelength (Å)	0.97
Space group	*I*4
No. of unique reflections	61537 (3673)
Resolution range (Å)	47.90–1.20 (1.23–1.20)
Multiplicity	5.34 (2.11)
〈*I*/σ(*I*)〉	12.23 (1.13)
*R*_merge_ (%)	6.30 (83.80)
CC_1/2_ (%)	99.80 (48.90)
*R*_p.i.m._ (%)	2.7 (64.6)
Data completeness (%)	97.40 (79.00)
Crystallographic refinement	
*R*_factor_ (%)	13.26
*R*_free_ (%)	14.77
Ramachandran plot	
Outliers (%)	0
Favored (%)	100
RMSD_bonds_ (Å)	0.004
RMSD_angles_ (°)	0.727
